# Refining Inaccurate Transmitter and Receiver Positions Using Calibration Targets for Target Localization in Multi-Static Passive Radar

**DOI:** 10.3390/s19153365

**Published:** 2019-07-31

**Authors:** Yongsheng Zhao, Dexiu Hu, Yongjun Zhao, Zhixin Liu, Chuang Zhao

**Affiliations:** PLA Strategic Support Force Information Engineering University, Zhengzhou 450001, China

**Keywords:** multi-static passive radar, target localization, calibration target, bistatic range, transmitter and receiver position error, Cramér–Rao lower bound

## Abstract

Transmitter and receiver position errors have been known to significantly deteriorate target localization accuracy in a multi-static passive radar (MPR) system. This paper explores the use of calibration targets, whose positions are known to the MPR system, to counter the loss in target localization accuracy arising from transmitter/receiver position errors. This paper firstly evaluates the Cramér–Rao lower bound (CRLB) for bistatic range (BR)-based target localization with calibration targets, which analytically indicates the potential of calibration targets in enhancing localization accuracy. After that, this paper proposes a novel closed-form solution, which includes two steps: calibration step and localization step. Firstly, the calibration step is devoted to refine the inaccurate transmitter and receiver locations using the BR measurements from the calibration targets, and then in the calibration step, the target localization can be accurately achieved by using the refined transmitter/receiver positions and the BR measurements from the unknown target. Theoretical analysis and simulation results indicate that the proposed method can attain the CRLB at moderate measurement noise level, and exhibits the superiority of localization accuracy over existing algorithms.

## 1. Introduction

Passive radar technology, which allows operators to detect and localize potential targets using already existing transmitters such as commercial frequency modulation (FM) broadcast/digital audio broadcast (DAB)/terrestrial digital video broadcast (DVB-T) [1,2] and non-cooperative radar transmission [3,4], has been interesting to both civilian and military fields in the last few decades [5]. This sort of radar, compared to active radar technology, offers numerous advantages including lower cost, lower power usage and more covert surveillance capability, which suggests the possibility of employing passive radars on a wide range of concerned applications such as homeland security, costal surveillance and early warning system for vehicles detection, etc.

One of the remarkable characters of passive radar is the deployment of two receiving channels, with one for capturing the direct path signal from the transmitter and the other for collecting the potential target echoes [6]. By performing a cross-correlation operation between the direct path signal and the target echoes, the time delay (TD) could be measured, which holds information about the unknown target position. By multiplying with the signal propagation speed, the TD can be directly converted into the bistatic range (BR) [7]. Each BR measurement traces out an ellipsoid equation, with its foci located at the transmitter and the receiver positions. Theoretically, for multi-static passive radar (MPR), if enough BR measurements with respect to multiple transmitter-receiver pairs are available, the target position can be determined by solving the set of nonlinear ellipsoid equations. However, due to the high nonlinearity implied in the BR measurement equations, determining the target position from the BR measurements obtained at a single time instant is not a trivial topic.

In recent years, numerous algorithms have been developed to address this challenging topic, mainly including iterative methods [8,9] and closed-form solution methods [10,11,12,13,14]. Iterative methods [8,9] rely on an initial position guess close to the true solution but such a good guess may not always be available in reality. By contrast, closed-form solution methods [10,11,12,13,14] have always been more attractive to researchers due to their computational efficiency, independence on initial guess and absence of divergence problem. Illuminated by Ho and Xu’s two-step weighted least squares (2WLS) idea [15], these closed-form solution methods [10,11,12,13,14] generally follow the basic two-step framework below: in the first step, the non-linear BR equations are linearized into pseudo-linear ones by introducing some proper nuisance parameters and a coarse estimate of target position can be obtained from the pseudo-linear equation set via weighted least squares (WLS) minimization; then in the second step, the function relation between the target position and the introduced nuisance parameters is explored to refine the initial estimate. 

Nevertheless, all the aforementioned methods are designed based on the assumption that the positions of the transmitters and receivers are exactly known, but such exact priori knowledge may not be available in reality. In fact, the positions of the transmitters and receivers are inevitably perturbed by errors to some extent, and these errors (also referred to as position uncertainties) are often non-negligible, especially when the antennas are mounted on moving platforms [16,17] or the exploited transmitters are highly non-cooperative (such as the hostile radar radiation whose position could usually only roughly determined by electronic reconnaissance technique [18,19]). On the other hand, Rui and Ho [20] quantitatively analyze the influence of the transmitter and receiver position error on the localization accuracy, indicating that the target localization accuracy can be very sensitive to the transmitter/receiver position error and a slight error in transmitter/receiver position could remarkably deteriorate the localization accuracy. More recently, some novel methods [21,22] that take the statistical distributions of transmitter/receiver position error into consideration are developed to reduce the target localization error, and they are shown to attain the Cramér–Rao lower bound (CRLB) under small measurement noise and transmitter/receiver position error assumption. Nevertheless, these methods [21,22] only present the solutions when the transmitter/receiver position errors exist, but cannot fundamentally compensate the localization performance loss arising from transmitter/receiver position error at the CRLB level. 

The use of calibration sensors has been a common technique in wireless sensor network self-localization, where each sensor broadcasts signals and receives signals from other sensors so as to determine their positions collaboratively [23,24]. Syldatk [25,26] considers the calibration of ground sensor networks where an accurate calibration of sensor positions and orientations is required for target tracking. For source localization problem, Hasan first considered in [27] utilizing calibration sensors to improve the angle-based source localization performance. Ho [28], Yang [29] as well as Li [30] et al. further expanded and applied the calibration technique to range difference (RD)-based source localization problem, where additional RD measurements from the calibration sensors were incorporated to reduce the receivers’ position error and thus improve the source localization accuracy. The successful use of calibration techniques in these fields inspires us the possibility of employing calibration technique in the target localization for multi-static passive radar. When it comes to BR-based target localization in multi-static passive radar, using a ‘calibration target’ with known position may also be able to mitigate the target localization performance loss arising from the transmitter/receiver position error. In theory, any target appearing in the radar coverage area and meanwhile broadcasting its position can be taken as a calibration target. Typically, for example, to avoid potential accidents and collisions, the commercial aircrafts will report their positions and other information to the ground stations and other aircraft by using the automatic dependent surveillance broadcast (ADS-B) system [31]. Hence, the commercial aircraft broadcasting ADS-B signal can be regarded as an off-the-shelf calibration target. If no such off-the-shelf calibration targets are available in the radar coverage area, we can manually launch some strong scatterers with known positions as calibration targets. However, despite that, up to now there exists no publication in the open literature that addresses refining inaccurate transmitter and receiver positions using calibration targets for target localization in multi-static passive radar.

Motivated by these facts, in this paper, taking the transmitter and receiver position error into consideration, we explore using calibration targets to counter the loss in BR-based target localization accuracy arising from the transmitter/receiver position error. We begin our work by deriving the CRLB for BR-based target position estimation when the BR measurements from the calibration targets are available. The interpretation on the CRLB demonstrates that the use of calibration targets can significantly mitigate the influence of the transmitter/receiver position error and dramatically enhance the localization accuracy, at least in the sense of CRLB. We then proceed to develop a novel localization method to alleviate the transmitter and receiver position error and enhance the localization accuracy using calibration targets. It mainly includes two processing stages, referred to as calibration stage and localization stage respectively. In the calibration stage, the BR measurements from the calibration targets are exploited to refine the inaccurate transmitter and receiver positions; in the localization stage, the refined transmitter and receiver positions and BR measurements corresponding to the unknown target are exploited to determine the target position. Both processing stages are closed-form, which brings the proposed method computational efficiency and high robustness. Furthermore, the accuracy of the proposed solution is shown analytically to reach the CRLB when the transmitters’/receivers’/calibration targets’ position errors and the BR measurement noises are sufficiently small. Simulations will be conducted to verify the effectiveness and superiority of the proposed solution over existing methods.

*Notations*: There will be a lot of notations throughout this paper. Without exception, vectors (matrices) are denoted by bold lower (upper) case letters, respectively. Also, notations ${\left(\cdot \right)}^{{}^{\circ}}$, ${\left(\cdot \right)}^{T}$, ‖ · ‖ , ${\left(\cdot \right)}^{-1}$, E(·), $O_{p\times q}$, $I_{p\times p}$, $0_{p\times 1}$, diag(·) and tr(·), represent the true value of a noisy or an estimated variable, transpose operation, Euclidean norm, inverse of matrix, statistical expectation, a *p*-by-*q* zero matrix, an identity matrix of size *p*, a *p*-by-1 zero vector, diagonal matrix and the trace of a matrix, respectively. 

The remainder of this paper is organized as follows. Section 2 is about the localization scenario in the presence of transmitter/receiver position error and calibration targets. In Section 3, the corresponding CRLB is evaluated, indicating the potential of calibration targets in improve localization accuracy. In Section 4, a closed-form solution is developed for target localization in the presence of transmitter/receiver position error and calibration targets, and theoretical performance analysis is also given. Section 5 describes the results of Monte Carlo simulations that compare the proposed solution with existing methods. Section 6 is the conclusion.

## 2. Problem Formulation

**Figure 1:**
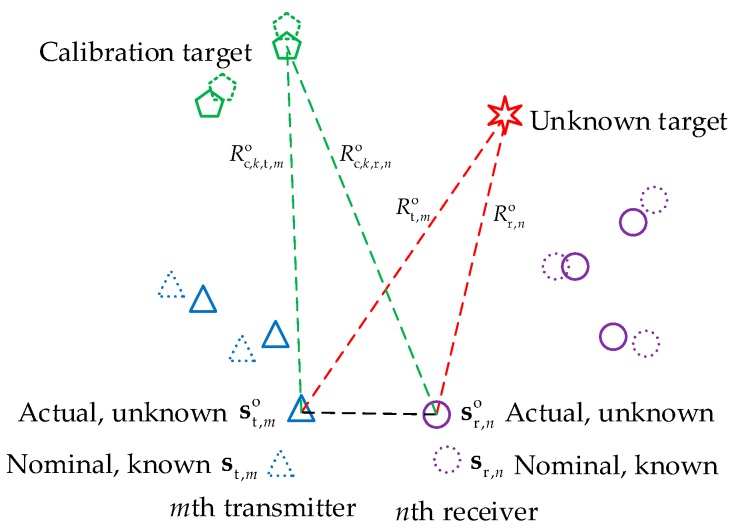
Practical scenario geometry of multi-static passive radar in the presence of transmitter/receiver position error and calibration targets.

Address a typical multi-static passive radar localization scenario as presented in Figure 1, where *M* non-cooperative transmitters located at $s_{t,m}^{o}={\left[x_{t,m}^{o},y_{t,m}^{o},z_{t,m}^{o}\right]}^{T}$ (m=1,2,…,M) are employed to illuminate the surveillance area, and *N* receivers located at $s_{r,n}^{o}={\left[x_{r,n}^{o},y_{r,n}^{o},z_{r,n}^{o}\right]}^{T}$ (n=1,2,…,N) are deployed to determine a single target’s position denoted by $u^{o}={\left[x^{o},y^{o},z^{o}\right]}^{T}$. In fact, the exact positions of the transmitters and receivers might not be known, and only the inaccurate measured versions, i.e., $s_{t,m}={\left[x_{t,m},y_{t,m},z_{t,m}\right]}^{T}$ and $s_{r,n}={\left[x_{r,n},y_{r,n},z_{r,n}\right]}^{T}$, are available for processing. Formulaically, we arrive at

(1) $$s_{t,m}=s_{t,m}^{o}+\Delta s_{t,m}$$

(2) $$s_{r,n}=s_{r,n}^{o}+\Delta s_{r,n}$$

where $\Delta s_{t,m}$ and $\Delta s_{r,n}$ are the position error of the *m*th transmitter and the *n*th receiver respectively and also referred to as position uncertainty. Stacking (1) and (2) with respect to all the transmitters and receivers, yields a 3(*M* + *N*)-by-1 transmitter and receiver position vector as

(3) $$s=s^{o}+\Delta s$$

where $s={\left[s_{t}^{T},s_{r}^{T}\right]}^{T}$ with $s_{t}={\left[s_{t,1}^{T},s_{t,2}^{T},\ldots ,s_{t,M}^{T}\right]}^{T}$ and $s_{r}={\left[s_{r,1}^{T},s_{r,2}^{T},\ldots ,s_{r,N}^{T}\right]}^{T}$ is the noisy transmitter and receiver position vector, $s^{o}={\left[{\left(s_{t}^{o}\right)}^{T},{\left(s_{r}^{o}\right)}^{T}\right]}^{T}$ with $s_{t}^{o}={\left[{\left(s_{t,1}^{o}\right)}^{T},{\left(s_{t,2}^{o}\right)}^{T},\ldots ,{\left(s_{t,M}^{o}\right)}^{T}\right]}^{T}$ and $s_{r}^{o}={\left[{\left(s_{r,1}^{o}\right)}^{T},{\left(s_{r,2}^{o}\right)}^{T},\ldots ,{\left(s_{r,N}^{o}\right)}^{T}\right]}^{T}$ is the true transmitter and receiver position vector, and $\Delta s={\left[\Delta s_{t}^{T},\Delta s_{r}^{T}\right]}^{T}$ with $\Delta s_{t}={\left[\Delta s_{t,1}^{T},\Delta s_{t,2}^{T},\ldots ,\Delta s_{t,M}^{T}\right]}^{T}$ and $\Delta s_{r}={\left[\Delta s_{r,1}^{T},\Delta s_{r,2}^{T},\ldots ,\Delta s_{r,N}^{T}\right]}^{T}$ is the transmitter and receiver position error vector that can be assumed zero-mean Gaussian with covariance $Q_{s}$ without loss of generality.

Using the above notations, the distance from the *m*th transmitter to the target of interest is equal to

(4) $$R_{t,m}^{o}=\|u^{o}-s_{t,m}^{o}\|$$

the distance from the target of interest to the *n*th receiver is equal to

(5) $$R_{r,n}^{o}=\|u^{o}-s_{r,n}^{o}\|$$

and the baseline distance with respect to the mth transmitter and nth receiver is

(6) $$R_{t,m,r,n}^{o}=\|s_{t,m}^{o}-s_{r,n}^{o}\|$$

According to this, the BR measurement with respect to the *m*th transmitter and *n*th receiver, i.e., the sum of the distances from the *m*th transmitter to the target and the target to the *n*th receiver, can be formulized as

(7) $$\begin{array}{l} r_{m,n}=r_{m,n}^{o}+\Delta r_{m,n}\\ \;=R_{t,m}^{o}+R_{r,n}^{o}-R_{t,m,r,n}^{o}+\Delta r_{m,n}\\ \;=\|u^{o}-s_{t,m}^{o}\|+\|u^{o}-s_{r,n}^{o}\|-\|s_{t,m}^{o}-s_{r,n}^{o}\|+\Delta r_{m,n} \end{array}$$

where $r_{m,n}^{o}=R_{t,m}^{o}+R_{r,n}^{o}-R_{t,m,r,n}^{o}$ represents the true BR with respect to the *m*th transmitter and *n*th receiver, $\Delta r_{m,n}$ is the BR measurement noise. Herein, it is important to emphasize that the time delay measurement comes from the cross correlation operation between the target signal and the direct path reference signal [7], and its error characteristics are not affected by the transmitter/receiver position error since the transmitter/receiver position error is not involved in the estimation of time delay. Thus, the BR measurement noise is only related to the time delay measurement noise, and not related to the transmitter/receiver position error. Obviously, there will be *MN* BR measurements to be produced with respect to the *M* transmitters and *N* receivers, which can be recast into a *MN*-by-1 vector as

(8) $$r=r^{o}+\Delta r$$

where $r={\left[r_{1}^{T},r_{2}^{T},\ldots ,r_{M}^{T}\right]}^{T}$ with $r_{m}={\left[r_{m,1},r_{m,2},\ldots ,r_{m,N}\right]}^{T}$ is the BR measurement vector, $r^{o}={\left[{\left(r_{1}^{o}\right)}^{T},{\left(r_{2}^{o}\right)}^{T},\ldots ,{\left(r_{M}^{o}\right)}^{T}\right]}^{T}$ with $r_{m}^{o}={\left[r_{m,1}^{o},r_{m,2}^{o},\ldots ,r_{m,N}^{o}\right]}^{T}$ is the true BR vector, and $\Delta r={\left[\Delta r_{1}^{T},\Delta r_{2}^{T},\ldots ,\Delta r_{M}^{T}\right]}^{T}$ with $\Delta r_{m}={\left[\Delta r_{m,1},\Delta r_{m,2},\ldots ,\Delta r_{m,N}\right]}^{T}$ is the BR measurement noise vector, which is usually assumed follow a Gaussian distribution with zero-mean and covariance $Q_{r}$.

As presented in Figure 1, to alleviate the transmitter/receiver position error and enhance localization accuracy, *K* calibration targets located at $c_{k}^{o}={\left[x_{c,k}^{o},y_{c,k}^{o},z_{c,k}^{o}\right]}^{T}$ (k=1,2,…,K) are employed, and the BRs among the calibration targets and the transmitter-receiver pairs are also measured. Similarly, the exact positions of the calibration targets are not known to us, and the nominal versions denoted by $c_{k}={\left[x_{c,k},y_{c,k},z_{c,k}\right]}^{T}$ (k=1,2,…,K) are given as

(9) $$c_{k}=c_{k}^{o}+\Delta c_{k}$$

where $\Delta c_{k}$ is the position error of the *k*th calibration target. Collecting (9) for all the *K* calibration targets forms a 3*K*-by-1 calibration target position vector as

(10) $$c=c^{o}+\Delta c$$

where $c={\left[c_{1}^{T},c_{2}^{T},\ldots ,c_{K}^{T}\right]}^{T}$ is the nominal calibration target position vector, $c^{o}={\left[{\left(c_{1}^{o}\right)}^{T},{\left(c_{2}^{o}\right)}^{T},\ldots ,{\left(c_{K}^{o}\right)}^{T}\right]}^{T}$ is the true calibration target position vector, and $\Delta c={\left[\Delta c_{1}^{T},\Delta c_{2}^{T},\ldots ,\Delta c_{K}^{T}\right]}^{T}$ is the calibration target position error vector that is usually supposed to obey Gaussian distribution with zero-mean and covariance $Q_{c}$. Herein, it should be pointed out that, the positions of calibration targets are generally considered to be more precise compared with those of the transmitters and receivers, although they are also contaminated by errors.

Then, the distance from the *m*th transmitter to the *k*th calibration target is given by

(11) $$R_{c,k,t,m}^{o}=\|c_{k}^{o}-s_{t,m}^{o}\|$$

and the distance from the *k*th calibration target to the *n*th receiver is given by

(12) $$R_{c,k,r,n}^{o}=\|c_{k}^{o}-s_{r,n}^{o}\|$$

Based on this, the BR measurement corresponding to the *k*th calibration target, *m*th transmitter and *n*th receiver can be modeled as

(13) $$\begin{array}{l} r_{c,k,m,n}=r_{c,k,m,n}^{o}+\Delta r_{c,k,m,n}\\ \;=R_{c,k,t,m}^{o}+R_{c,k,r,n}^{o}-R_{t,m,r,n}^{o}+\Delta r_{c,k,m,n}\\ \;=\|c_{k}^{o}-s_{t,m}^{o}\|+\|c_{k}^{o}-s_{r,n}^{o}\|-\|s_{t,m}^{o}-s_{r,n}^{o}\|+\Delta r_{c,k,m,n} \end{array}$$

where $\Delta r_{c,k,m,n}$ represents measurement noise in $r_{c,k,m,n}$, $r_{c,k,m,n}^{o}=R_{c,k,t,m}^{o}+R_{c,k,r,n}^{o}-R_{t,m,r,n}^{o}$ represents the true BR with respect to the *k*th calibration target, *m*th transmitter and *n*th receiver. Collecting (13) for the set of *K* calibration targets, *M* transmitters and *N* receivers, results in a *KMN*-by-1 vector as

(14) $$r_{c}=r_{c}^{o}+\Delta r_{c}$$

where $r_{c}={\left[r_{c,1}^{T},r_{c,2}^{T},\ldots ,r_{c,K}^{T}\right]}^{T}$ with $r_{c,k}={\left[r_{c,k,1}^{T},r_{c,k,2}^{T},\ldots ,r_{c,k,M}^{T}\right]}^{T}$ and $r_{c,k,m}={\left[r_{c,k,m,1},r_{c,k,m,2},\ldots ,r_{c,k,m,N}\right]}^{T}$ denotes the BR measurement vector from the calibration targets, $r_{c}^{o}={\left[{\left(r_{c,1}^{o}\right)}^{T},{\left(r_{c,2}^{o}\right)}^{T},\ldots ,{\left(r_{c,K}^{o}\right)}^{T}\right]}^{T}$ with $r_{c,k}^{o}={\left[{\left(r_{c,k,1}^{o}\right)}^{T},{\left(r_{c,k,2}^{o}\right)}^{T},\ldots ,{\left(r_{c,k,M}^{o}\right)}^{T}\right]}^{T}$ and $r_{c,k,m}^{o}={\left[r_{c,k,m,1}^{o},r_{c,k,m,2}^{o},\ldots ,r_{c,k,m,N}^{o}\right]}^{T}$ denotes the corresponding true value vector, $\Delta r_{c}={\left[\Delta r_{c,1}^{T},\Delta r_{c,2}^{T},\ldots ,\Delta r_{c,K}^{T}\right]}^{T}$ with $\Delta r_{c,k}={\left[\Delta r_{c,k,1}^{T},\Delta r_{c,k,2}^{T},\ldots ,\Delta r_{c,k,M}^{T}\right]}^{T}$ and $\Delta r_{c,k,m}={\left[\Delta r_{c,k,m,1},\Delta r_{c,k,m,2},\ldots ,\Delta r_{c,k,m,N}\right]}^{T}$ denotes the corresponding error vector, which is presumed to be a Gaussian random vector with zero mean and covariance $Q_{rc}$.

Now, the purpose of this work is to determine the target position from the noisy BR measurements and the inaccurate transmitter/receiver positions. In particular, the calibration targets with known position and the corresponding BR measurements are also available to reduce the transmitter/receiver position error and improve localization accuracy.

## 3. Evaluation of the CRLB with Calibration Targets

The CRLB does not address the specific estimators employed, but simply reflects minimum possible variance that an unbiased estimator can achieve with existing observations. In this section, in order to justify the necessity of refining the inaccurate transmitter and receiver positions using calibration targets, we shall first set up the CRLB for the target localization problem described above. Besides the BR measurement noise, the position errors of transmitter, receivers and calibration targets are also included. From the localization scenario presented in Section 2, the deterministic but unknown parameters for the CRLB evaluation, collected into a 3(*M* + *N* + *K* + 1)-by-1 vector $\varphi ={\left[{\left(u^{o}\right)}^{T},{\left(s^{o}\right)}^{T},{\left(c^{o}\right)}^{T}\right]}^{T}$, include the target position vector $u^{o}$, the transmitter and receiver position vector $s^{o}$, and the calibration target position vector $c^{o}$; the observations, collected into a (*MN* + *KMN* + 3*M* + 3*N* + 3*K*)-by-1 vector $z={\left[r^{T},r_{c}^{T},s^{T},c^{T}\right]}^{T}$, include the BR measurement vector **r** from the unknown target, the BR measurement vector $r_{c}$ from the calibration targets, the inaccurate measured transmitter and receiver position vector **s**, and the nominal calibration target position vector **c**, which are Gaussian distributed and independent with one another. Based on this, the joint probability density function (pdf) of the observations parameterized by the unknown parameter vector is readily shown to be

(15) $$\begin{array}{l} p(z|\varphi )=p(r|u^{o},s^{o})\cdot p(r_{c}|s^{o},c^{o})\cdot p(s|s^{o})\cdot p(c|c^{o})\\ \;=\kappa \cdot \exp[-\frac{1}{2}{\left(r-r^{o}\right)}^{T}Q_{r}^{-1}(r-r^{o})-\frac{1}{2}{\left(r_{c}-r_{c}^{o}\right)}^{T}Q_{rc}^{-1}(r_{c}-r_{c}^{o})\\ \;-\frac{1}{2}{\left(s-s^{o}\right)}^{T}Q_{s}^{-1}(s-s^{o})-\frac{1}{2}{\left(c-c^{o}\right)}^{T}Q_{c}^{-1}(c-c^{o})]\; \end{array}$$

where κ is a constant with respect to the unknown parameters. By taking the logarithm of (15), partial derivatives with respect to the unknown parameters twice, and then expectation, the Fisher information matrix (FIM) can be calculated as

(16) $$\begin{array}{l} FIM(\varphi )=E\left[\frac{\partial \ln p(z|\varphi )}{\partial \varphi }\left(\frac{\partial \ln p(z|\varphi )}{\partial \varphi }\right)\right]\\ \;=\left[\begin{array}{ccc} X & Y & O_{3\times 3}\\ Y^{T} & Z & R^{T}\\ O_{3\times 3} & R & P \end{array}\right] \end{array}$$

where the blocks **X**, **Y**, **Z**, **R** and **P** are respectively given by

(17) $$X={\left(\frac{\partial r^{o}}{\partial u^{o}}\right)}^{T}Q_{r}^{-1}\left(\frac{\partial r^{o}}{\partial u^{o}}\right)$$

(18) $$Y={\left(\frac{\partial r^{o}}{\partial u^{o}}\right)}^{T}Q_{r}^{-1}\left(\frac{\partial r^{o}}{\partial s^{o}}\right)$$

(19) $$Z=Q_{s}^{-1}+{\left(\frac{\partial r^{o}}{\partial s^{o}}\right)}^{T}Q_{r}^{-1}\left(\frac{\partial r^{o}}{\partial s^{o}}\right)+{\left(\frac{\partial r_{c}^{o}}{\partial s^{o}}\right)}^{T}Q_{rc}^{-1}\left(\frac{\partial r_{c}^{o}}{\partial s^{o}}\right)$$

(20) $$R={\left(\frac{\partial r_{c}^{o}}{\partial c^{o}}\right)}^{T}Q_{rc}^{-1}\left(\frac{\partial r_{c}^{o}}{\partial s^{o}}\right)$$

(21) $$P=Q_{c}^{-1}+{\left(\frac{\partial r_{c}^{o}}{\partial c^{o}}\right)}^{T}Q_{rc}^{-1}\left(\frac{\partial r_{c}^{o}}{\partial c^{o}}\right)$$

Denote $i_{m,n}=(m-1)N+n$, and $i_{k,m,n}=(k-1)MN+(m-1)N+n$. From the formulations of (7) and (13), the elements of the partial derivatives $\partial r^{o}/\partial u^{o}$, $\partial r^{o}/\partial s^{o}$, $\partial r_{c}^{o}/\partial c^{o}$ and $\partial r_{c}^{o}/\partial s^{o}$ in (17)–(21), can be determined as

(22) $$\frac{\partial r^{o}}{\partial u^{o}}(i_{m,n},1:3)=\frac{{\left(u^{o}-s_{t,m}^{o}\right)}^{T}}{R_{t,m}^{o}}+\frac{{\left(u^{o}-s_{r,n}^{o}\right)}^{T}}{R_{r,n}^{o}}$$

(23) $$\frac{\partial r^{o}}{\partial s^{o}}=\left[\begin{array}{cc} \frac{\partial r^{o}}{\partial s_{t}^{o}} & \frac{\partial r^{o}}{\partial s_{r}^{o}} \end{array}\right]$$

(24) $$\frac{\partial r^{o}}{\partial s_{t}^{o}}(i_{m,n},3m-2:3m)=\frac{{\left(s_{t,m}^{o}-u^{o}\right)}^{T}}{R_{t,m}^{o}}-\frac{{\left(s_{t,m}^{o}-s_{r,n}^{o}\right)}^{T}}{R_{t,m,r,n}^{o}}$$

(25) $$\frac{\partial r^{o}}{\partial s_{r}^{o}}(i_{m,n},3n-2:3n)=\frac{{\left(s_{r,n}^{o}-u^{o}\right)}^{T}}{R_{r,n}^{o}}-\frac{{\left(s_{r,n}^{o}-s_{t,m}^{o}\right)}^{T}}{R_{t,m,r,n}^{o}}$$

(26) $$\frac{\partial r_{c}^{o}}{\partial c^{o}}(i_{k,m,n},3k-2:3k)=\frac{{\left(c_{k}^{o}-s_{t,m}^{o}\right)}^{T}}{R_{c,k,t,m}^{o}}+\frac{{\left(c_{k}^{o}-s_{r,n}^{o}\right)}^{T}}{R_{c,k,r,n}^{o}}$$

(27) $$\frac{\partial r_{c}^{o}}{\partial s^{o}}=\left[\begin{array}{cc} \frac{\partial r_{c}^{o}}{\partial s_{t}^{o}} & \frac{\partial r_{c}^{o}}{\partial s_{r}^{o}} \end{array}\right]$$

(28) $$\frac{\partial r_{c}^{o}}{\partial s_{t}^{o}}(i_{k,m,n},3m-2:3m)=\frac{{\left(s_{t,m}^{o}-c_{k}^{o}\right)}^{T}}{R_{c,k,t,m}^{o}}-\frac{{\left(s_{t,m}^{o}-s_{r,n}^{o}\right)}^{T}}{R_{t,m,r,n}^{o}}$$

(29) $$\frac{\partial r_{c}^{o}}{\partial s_{r}^{o}}(i_{k,m,n},3n-2:3n)=\frac{{\left(s_{r,n}^{o}-c_{k}^{o}\right)}^{T}}{R_{c,k,r,n}^{o}}-\frac{{\left(s_{r,n}^{o}-s_{t,m}^{o}\right)}^{T}}{R_{t,m,r,n}^{o}}$$

for k=1,2,…,K, m=1,2,…,M and n=1,2,…,N, and zeros elsewhere.

By definition, the CRLB of φ, denoted by $CRLB_{c}(\varphi )$, is given as $\mathrm{FIM}{\left(\varphi \right)}^{-1}$, where only the upper left 3-by-3 block is for the target position $u^{o}$. Invoking the partitioned matrix inversion formula as well as the matrix inversion lemma [32] twice on (16), leads to the CRLB of $u^{o}$ as

(30) $$CRLB_{c}(u^{o})=X^{-1}+X^{-1}Y{\left(Z-Y^{T}X^{-1}Y-R^{T}P^{-1}R\right)}^{-1}Y^{T}X^{-1}$$

For comparison purposes, the CRLB of $u^{o}$ with transmitter/receiver position error but without calibration derived in [22], denoted by $CRLB_{s}(u^{o})$, is also given below

(31) $$CRLB_{s}(u^{o})=X^{-1}+X^{-1}Y{\left(\overset{⌢}{Z}-Y^{T}X^{-1}Y\right)}^{-1}Y^{T}X^{-1}$$

where $\overset{⌢}{Z}=Q_{s}^{-1}+{\left(\partial r^{o}/\partial s^{o}\right)}^{T}Q_{r}^{-1}(\partial r^{o}/\partial s^{o})$. For the sake of comparison, we proceed to construct an equivalent form of $CRLB_{c}(u^{o})$ by denoting $\overset{⌣}{Z}$ as $\overset{⌣}{Z}=Z-R^{T}P^{-1}R$. After invoking the matrix inversion lemma [32] to ${\left(\partial r_{c}^{o}/\partial s^{o}\right)}^{T}Q_{rc}^{-1}(\partial r_{c}^{o}/\partial s^{o})-R^{T}P^{-1}R$ and some algebraic manipulations, we further represent $\overset{⌣}{Z}$ as

(32) $$\overset{⌣}{Z}=Q_{s}^{-1}+{\left(\frac{\partial r^{o}}{\partial s^{o}}\right)}^{T}Q_{r}^{-1}\left(\frac{\partial r^{o}}{\partial s^{o}}\right)+{\left(\frac{\partial r_{c}^{o}}{\partial s^{o}}\right)}^{T}{\left(Q_{rc}+\left(\frac{\partial r_{c}^{o}}{\partial c^{o}}\right)Q_{c}{\left(\frac{\partial r_{c}^{o}}{\partial c^{o}}\right)}^{T}\right)}^{-1}\left(\frac{\partial r_{c}^{o}}{\partial s^{o}}\right)$$

Using (32), we obtain an equivalent expression of $CRLB_{c}(u^{o})$ as

(33) $$CRLB_{c}(u^{o})=X^{-1}+X^{-1}Y{\left(\overset{⌣}{Z}-Y^{T}X^{-1}Y\right)}^{-1}Y^{T}X^{-1}$$

Through the comparison of (31) and (33), it is readily to observe that the two CRLBs are identical in structure, except that $\overset{⌣}{Z}$ is substituted by $\overset{⌢}{Z}$. More specifically, the use of calibration targets introduces an additional component into the bracketed matrix expression to be inverted as

(34) $$\begin{array}{l} \tilde{Z}=\overset{⌣}{Z}-\overset{⌢}{Z}\\ \;={\left(\partial r_{c}^{o}/\partial s^{o}\right)}^{T}Q_{rc}^{-1}(\partial r_{c}^{o}/\partial s^{o})-R^{T}P^{-1}R\\ \;={\left(\partial r_{c}^{o}/\partial s^{o}\right)}^{T}{\left(Q_{rc}+(\partial r_{c}^{o}/\partial c^{o})Q_{c}{\left(\partial r_{c}^{o}/\partial c^{o}\right)}^{T}\right)}^{-1}(\partial r_{c}^{o}/\partial s^{o}) \end{array}$$

Using (34), we can rewrite ${\left(\overset{⌣}{Z}-Y^{T}X^{-1}Y\right)}^{-1}$ in (33) as ${\left((\overset{⌢}{Z}-Y^{T}X^{-1}Y)+\tilde{Z}\right)}^{-1}$. Invoking the matrix inversion lemma [32] to the term ${\left((\overset{⌢}{Z}-Y^{T}X^{-1}Y)+\tilde{Z}\right)}^{-1}$ in (33), we obtain after some algebraic manipulations,

(35) $$CRLB_{c}(u^{o})-CRLB_{s}(u^{o})=X^{-1}Y\Gamma Y^{T}X^{-1}$$

where

(36) $$\Gamma ={Η}^{-1}ϒ{\left(I+{ϒ}^{T}{Η}^{-1}ϒ\right)}^{-1}{ϒ}^{T}{Η}^{-1}$$

(37) $$Η=(\tilde{Z}-Y^{T}X^{-1}Y)$$

(38) $$ϒ={\left(\frac{\partial r_{c}^{o}}{\partial s^{o}}\right)}^{T}L_{rc}$$

and $L_{rc}$ is the Cholesky decomposition of ${\left(Q_{rc}+(\partial r_{c}^{o}/\partial c^{o})Q_{c}{\left(\partial r_{c}^{o}/\partial c^{o}\right)}^{T}\right)}^{-1}$, i.e., $L_{rc}L_{rc}^{T}={\left(Q_{rc}+(\partial r_{c}^{o}/\partial c^{o})Q_{c}{\left(\partial r_{c}^{o}/\partial c^{o}\right)}^{T}\right)}^{-1}$. In form, the right side of (35) is just the performance enhancement because of the use of calibration targets. It is positive semi-definite (PSD) since it has a symmetric structure and ${ϒ}^{T}$ is not full column rank. Even if the nominal positions of calibration targets and the corresponding BR measurements are very noisy, (35) can still remain PSD. In theory, only in the edge case when ${\left(Q_{rc}+(\partial r_{c}^{o}/\partial c^{o})Q_{c}^{-1}{\left(\partial r_{c}^{o}/\partial c^{o}\right)}^{T}\right)}^{-1}$ tends to zero and then $L_{rc}\to O$ and ϒ→O, the performance enhancement in (34) would tend to zero. However, this edge case hardly exists in reality. Thus, mathematically, we can arrive at

(39) $$CRLB_{s}(u^{o})\geq CRLB_{c}(u^{o})$$

The matrix inequality A≥B means that A−B is PSD. It can be further deduced from (39) that $\mathrm{tr}(CRLB_{s}(u^{o}))\geq \mathrm{tr}(CRLB_{c}(u^{o}))$. The trace of $CRLB_{c}(u^{o})$ and $CRLB_{s}(u^{o})$ respectively represents minimum possible variance of target position estimation with and without using calibration targets. Therefore, we can conclude that using calibration targets brings potential enhancement to the target localization accuracy, at least at the CRLB level.

**Figure 2:**
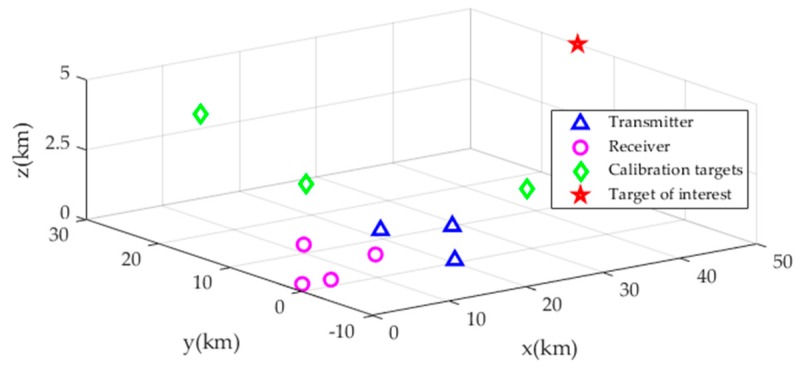
Localization scenario geometry for simulation.

**Figure 3:**
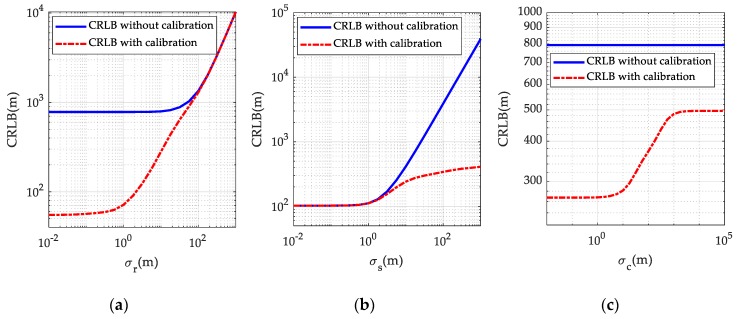
Comparison of the CRLBs with and without using calibration targets: (**a**) for different BR measurement noise level $\sigma_{r}$; (**b**) for different transmitter/receiver position error level $\sigma_{s}$; (**c**) for different calibration target position error level $\sigma_{c}$.

*Example* 1. To substantiate the evaluation on the CRLB presented above, a numerical example using a typical multi-static passive radar localization scenario was conducted, as presented in Figure 2. There are *M* = 3 transmitters, *N* = 4 receivers and *K* = 3 calibration targets in the scenario, and their true positions are listed in Table 1. The noise covariance matrix of the BR measurements from the unknown target are given by $Q_{r}=\sigma_{r}^{2}V_{r}$, where $\sigma_{r}$ reflects BR measurement noise level and $V_{r}$ is set to 1 in the diagonal elements and 0.5 elsewhere. The covariance matrix of the transmitter/receiver position error is given as $Q_{s}=\sigma_{s}^{2}V_{s}$ where $\sigma_{s}$ reflects the transmitter/receiver position error level and $V_{s}=\mathrm{diag}(5I_{3M\times 3M},I_{3N\times 3N})$. The covariance matrix of calibration target position error is $Q_{c}=\sigma_{c}^{2}V_{c}$ where $\sigma_{c}$ reflects the calibration target position error level and $V_{c}=I_{3K\times 3K}$, and the covariance matrix of the corresponding calibration BR measurement noise is $Q_{rc}=\sigma_{\mathrm{rc}}^{2}V_{rc}$ where $\sigma_{\mathrm{rc}}=\sigma_{r}$ reflects calibration BR measurement noise level and $V_{rc}$ is set to 1 in the diagonal elements and 0.5 elsewhere. The target of interest is located at position $u^{o}={\left[50000,15000,5000\right]}^{T}m$. The effect of the calibration targets on the target localization accuracy, in the sense of CRLB, is presented in Figure 3.

Figure 3a compares the CRLB curves with and without using calibration targets when the BR measurement noise level $\sigma_{r}$ is varied from $10^{-2}\;m$ to $10^{3}\;m$ while the transmitter/receiver position error level and calibration target position error level are fixed at $\sigma_{s}=20\;m$ and $\sigma_{c}=10\;m$ respectively. It can be observed from Figure 3a that the CRLB with calibration targets is generally below the one without, this coincides with the analytical conclusion given in (39). However, in the edge case where the BR measurement noise is very large, two CRLBs would tend to be the same. This is because in this case, the BR measurement noise dominates and effect of transmitter/receiver position error on the localization accuracy is relatively small. The CRLB curves versus the transmitter/receiver position error level $\sigma_{s}$ are plotted in Figure 3b where the BR measurement noise level and calibration target position error level are fixed at $\sigma_{r}=10\;m$ and $\sigma_{c}=10\;m$ respectively. A similar trend, i.e., two CRLBs would tend to be the same, appears in Figure 3b, when the transmitter/receiver position error is sufficiently small. A reasonable explanation is that, in this case, the transmitter/receiver positions are known very accurately and their influence on the localization accuracy can be ignored compared to the BR measurement noise. The CRLB comparison versus calibration target position error level $\sigma_{c}$ is provided in Figure 3c where $\sigma_{r}=10\;m$ and $\sigma_{s}=20\;m$. Interestingly, the trend of CRLB curves implies that, even when the calibration target position error is extremely large, the CRLB with utilization of calibration targets are still remarkably below the one without. This justifies again the analysis under (35), and similar results have also been presented in previous studies [23,24,25,26,27,28,29,30] on source localization and sensor network localization issues. Generally, from Figure 3, the use of calibration targets brings a significant improvement in the localization accuracy in the normal case, at least at the CRLB level.

## 4. Proposed Localization Method

The evaluation of the CRLB in Section 3 has demonstrated the potential of calibration targets in improving localization accuracy. In what follows, we will proceed to develop a novel closed-form solution for the aforementioned practical localization scenario where the positions of transmitters and receivers are inaccurate but calibration targets are used to refine the transmitter/receiver position and enhance the localization accuracy. After that, a theoretical analysis will be performed to show that the proposed solution achieves the CRLB when satisfying some mild conditions.

### 4.1. Algorithm Development

The proposed solution mainly includes two processing stages, referred to as calibration stage and localization stage, respectively. The calibration stage is devoted to refining the inaccurate transmitter and receiver positions, and then the localization stage is devoted to determining the target position on the basis of the refined transmitter and receiver positions.

#### 4.1.1. Calibration Stage

To make use of the BR measurements from the calibration targets, the calibration stage begins by reorganizing (13) as

(40) $$r_{c,k,m,n}-\|c_{k}^{o}-s_{t,m}^{o}\|-\|c_{k}^{o}-s_{r,n}^{o}\|+\|s_{t,m}^{o}-s_{r,n}^{o}\|=\Delta r_{c,k,m,n}$$

Since only the erroneous versions of $c_{k}^{o}$, $s_{t,m}^{o}$ and $s_{r,n}^{o}$ are available, we put $c_{k}^{o}=c_{k}-\Delta c_{k}$, $s_{t,m}^{o}=s_{t,m}-\Delta s_{t,m}$ and $s_{r,n}^{o}=s_{r,n}-\Delta s_{r,n}$ into (40), and then expand it around erroneous values $c_{k}$, $s_{t,m}$ and $s_{r,n}$ to the linear error terms as

(41) $$\begin{array}{l} r_{c,k,m,n}-\;\|c_{k}-s_{t,m}\;\|-\;\|c_{k}-s_{r,n}\;\|+\|s_{t,m}-s_{r,n}\|-(\rho_{c,k,t,m}^{T}+\rho_{t,m,r,n}^{T})\Delta s_{t,m}-(\rho_{c,k,r,n}^{T}-\rho_{t,m,r,n}^{T})\Delta s_{r,n}\\ =-{\left(\rho_{c,k,t,m}+\rho_{c,k,r,n}\right)}^{T}\Delta c_{k}+\Delta r_{c,k,m,n} \end{array}$$

where

(42) $$\rho_{c,k,t,m}=\frac{c_{k}-s_{t,m}}{\left\|c_{k}-s_{t,m}\right\|}$$

(43) $$\rho_{c,k,r,n}=\frac{c_{k}-s_{r,n}}{\left\|c_{k}-s_{r,n}\right\|}$$

(44) $$\rho_{t,m,r,n}=\frac{s_{t,m}-s_{r,n}}{\left\|s_{t,m}-s_{r,n}\right\|}$$

Stacking (41) for all the *k*, *m* and *n*, we can formulate them in matrix form as

(45) $$h_{0}-G_{0}\Delta s=\Delta h_{0}$$

The elements of $h_{0}$, $G_{0}$ and $\Delta h_{0}$ are given by

(46) $$h_{0}(i_{k,m,n},1)=r_{c,k,m,n}-\;\|c_{k}-s_{t,m}\|-\|c_{k}-s_{r,n}\|+\|s_{t,m}-s_{r,n}\|$$

(47) $$G_{0}=\left[\begin{array}{cc} G_{0,t} & G_{0,r} \end{array}\right],G_{0,t}(i_{k,m,n},3m-2:3m)=\rho_{c,k,t,m}^{T}+\rho_{t,m,r,n}^{T},G_{0,r}(i_{k,m,n},3n-2:3n)=\rho_{c,k,r,n}^{T}-\rho_{t,m,r,n}^{T}$$

(48) $$G_{c}(i_{k,m,n},3k-2:3k)=-{\left(\rho_{c,k,t,m}+\rho_{c,k,r,n}\right)}^{T}$$

(49) $$\Delta h_{0}(i_{k,m,n},1)=-{\left(\rho_{c,k,t,m}+\rho_{c,k,r,n}\right)}^{T}\Delta c_{k}+\Delta r_{c,k,m,n}$$

for $i_{k,m,n}=(k-1)MN+(m-1)N+n$, k=0,1,…,K−1, m=0,1,…,M−1, n=0,1,…,N−1, and zeros elsewhere. Furthermore, the error vector $\Delta h_{0}$ can be recast using a compact representation as follows

(50) $$\Delta h_{0}=G_{c}\Delta c+\Delta r_{c}$$

from which we have the mean $E(\Delta h_{0})=0_{KMN\times 1}$ and the covariance $\mathrm{cov}(\Delta h_{0})=G_{c}Q_{c}G_{c}^{T}+Q_{rc}$. In (45), Δs represents the difference between the true and the nominal transmitter/receiver positions.

In order to refine the transmitter and receiver positions, Δs shall be estimated as accurately as possible. Recall that Δs is a Gaussian distributed random vector with mean $E(\Delta s)=0_{3(M+N)\times 1}$ and covariance matrix $\mathrm{cov}(\Delta s)=Q_{s}$, and it is independent of the error vector $\Delta h_{0}$. Thus according to the Bayesian Gauss–Markov theorem [33], the linear minimum mean square error (LMMSE) estimate of Δs can be obtained from (45) as

(51) $$\begin{array}{l} \Delta \hat{s}=E(\Delta s)+{\left(\mathrm{cov}{\left(\Delta s\right)}^{-1}+G_{0}^{T}\mathrm{cov}{\left(\Delta h_{0}\right)}^{-1}G_{s}\right)}^{-1}G_{0}^{T}\mathrm{cov}{\left(\Delta h_{0}\right)}^{-1}\left(h_{0}-G_{0}E(\Delta s)\right)\\ \;={\left(Q_{s}^{-1}+G_{0}^{T}{\left(G_{c}Q_{c}G_{c}^{T}+Q_{rc}\right)}^{-1}G_{0}\right)}^{-1}G_{0}^{T}{\left(G_{c}Q_{c}G_{c}^{T}+Q_{rc}\right)}^{-1}h_{0} \end{array}$$

Under the assumption that the noise in $G_{c}$ and $G_{0}$ is sufficiently small to be ignored, the covariance matrix of $\Delta \hat{s}$ can be given as

(52) $$\mathrm{cov}(\Delta s-\Delta \hat{s})={\left(Q_{s}^{-1}+G_{0}^{T}{\left(G_{c}Q_{c}G_{c}^{T}+Q_{rc}\right)}^{-1}G_{0}\right)}^{-1}$$

Using the estimate of transmitter and receiver position error in (51), we can refine the transmitter and receiver positions as

(53) $$\hat{s}=s-\Delta \hat{s}$$

Utilizing the fact $s=s^{o}+\Delta s$, we can rewrite $\hat{s}$ in (53) as $\hat{s}=s^{o}+\Delta s-\Delta \hat{s}$. Hence, the refined estimate of transmitter/receiver positions $\hat{s}$ has a covariance matrix identical with (52). Forming the inverse of $\mathrm{cov}(\Delta s-\Delta \hat{s})$ and then comparing it to $Q_{s}^{-1}$ results in $\mathrm{cov}{\left(\Delta s-\Delta \hat{s}\right)}^{-1}-Q_{s}^{-1}=G_{0}^{T}{\left(G_{c}Q_{c}G_{c}^{T}+Q_{rc}\right)}^{-1}G_{0}$. It is natural to deduce that $\mathrm{cov}{\left(\Delta s-\Delta \hat{s}\right)}^{-1}\geq Q_{s}^{-1}$ is PSD since $G_{0}^{T}{\left(G_{c}Q_{c}G_{c}^{T}+Q_{rc}\right)}^{-1}G_{0}$ has a symmetric structure and $G_{c}$ is not full column rank. According to the PSD matrix property [34], $\mathrm{cov}{\left(\Delta s-\Delta \hat{s}\right)}^{-1}\geq Q_{s}^{-1}$ is equivalent to $Q_{s}\geq \mathrm{cov}(\Delta s-\Delta \hat{s})$. That is to say, the refined positions of transmitters and receivers performs leastwise as well as, if not better than, the original ones, in terms of target localization accuracy.

#### 4.1.2. Localization Stage

The localization stage starts by linearizing the BR equations from the unknown target. Firstly, reorganize (7) as 

(54) $$(r_{m,n}+R_{t,m,r,n}^{o})-R_{t,m}^{o}=R_{r,n}^{o}+\Delta r_{m,n}$$

Since we have obtained the refined estimate of $s_{t,m}^{o}$ and $s_{r,n}^{o}$ from calibration stage, we plug $s_{t,m}^{o}={\hat{s}}_{t,m}-(\Delta s_{t,m}-\Delta {\hat{s}}_{t,m})$ and $s_{r,n}^{o}={\hat{s}}_{r,n}-(\Delta s_{r,n}-\Delta {\hat{s}}_{r,n})$ into (54) and ignoring the second and higher error terms as

(55) $$\begin{array}{l} 2{\left({\hat{s}}_{t,m}-{\hat{s}}_{r,n}\right)}^{T}u^{o}+2(r_{m,n}+R_{t,m,r,n})R_{t,m}^{o}={\left(r_{m,n}+R_{t,m,r,n}\right)}^{2}+{\hat{s}}_{t,m}^{T}{\hat{s}}_{t,m}-{\hat{s}}_{r,n}^{T}{\hat{s}}_{r,n}-2R_{r,n}^{o}\Delta r_{m,n}\\ +2{\left(u^{o}-{\hat{s}}_{t,m}-R_{r,n}^{o}\rho_{t,m,r,n}\right)}^{T}(\Delta s_{t,m}-\Delta {\hat{s}}_{t,m})-2{\left(u^{o}-{\hat{s}}_{r,n}-R_{r,n}^{o}\rho_{t,m,r,n}\right)}^{T}(\Delta s_{r,n}-\Delta {\hat{s}}_{r,n}) \end{array}$$

By forming an auxiliary vector as $\theta^{o}={\left[{\left(u^{o}\right)}^{T},R_{t,1}^{o},R_{t,2}^{o},\ldots ,R_{t,M}^{o}\right]}^{T}$, we can collect (55) for all the *m* and *n* into a matrix form as

(56) $$G_{1}\theta^{o}=h_{1}+\Delta h_{1}$$

where

(57) $$G_{1}=\left[\begin{array}{cc} G_{1,s} & G_{1,r} \end{array}\right]$$

(58) $$G_{1,s}=2\left[\begin{array}{c} {\hat{s}}_{1}\\ {\hat{s}}_{2}\\ \vdots \\ {\hat{s}}_{M} \end{array}\right],s_{m}=\left[\begin{array}{c} {\left({\hat{s}}_{t,m}-{\hat{s}}_{r,1}\right)}^{T}\\ {\left({\hat{s}}_{t,m}-{\hat{s}}_{r,2}\right)}^{T}\\ \vdots \\ {\left({\hat{s}}_{t,m}-{\hat{s}}_{r,N}\right)}^{T} \end{array}\right],$$

(59) $$G_{1,r}=2\left[\begin{array}{cccc} r_{1} & 0_{N\times 1} & \cdots & 0_{N\times 1}\\ 0_{N\times 1} & r_{2} & \cdots & 0_{N\times 1}\\ \vdots & \vdots & \ddots & \vdots \\ 0_{N\times 1} & 0_{N\times 1} & \cdots & r_{M} \end{array}\right],r_{m}=\left[\begin{array}{c} r_{m,1}\\ r_{m,2}\\ \vdots \\ r_{m,N} \end{array}\right]$$

(60) $$h_{1}=\left[\begin{array}{c} h_{1,1}\\ h_{1,2}\\ \vdots \\ h_{1,M} \end{array}\right],h_{1,m}=\left[\begin{array}{c} {\left(r_{m,1}+R_{t,m,r,1}\right)}^{2}+{\hat{s}}_{t,m}^{T}{\hat{s}}_{t,m}-{\hat{s}}_{r,1}^{T}{\hat{s}}_{r,1}\\ {\left(r_{m,2}+R_{t,m,r,2}\right)}^{2}+{\hat{s}}_{t,m}^{T}{\hat{s}}_{t,m}-{\hat{s}}_{r,2}^{T}{\hat{s}}_{r,2}\\ \vdots \\ {\left(r_{m,N}+R_{t,m,r,N}\right)}^{2}+{\hat{s}}_{t,m}^{T}{\hat{s}}_{t,m}-{\hat{s}}_{r,N}^{T}{\hat{s}}_{r,N} \end{array}\right]$$

and the error vector $\Delta h_{1}$ is related to the target position as

(61) $$\Delta h_{1}=B_{1}\Delta r+D_{1}(\Delta s-\Delta \hat{s})$$

where

(62) $$B_{1}=\left[\begin{array}{cccc} B_{1,1} & O_{N\times N} & \cdots & O_{N\times N}\\ O_{N\times N} & B_{1,2} & \cdots & O_{N\times N}\\ \vdots & \vdots & \ddots & \vdots \\ O_{N\times N} & O_{N\times N} & \cdots & B_{1,M} \end{array}\right]$$

(63) $$D_{1}=\left[\begin{array}{ccccc} D_{1,t,1} & O_{N\times 3} & \cdots & O_{N\times 3} & D_{1,r,1}\\ O_{N\times 3} & D_{1,t,2} & \cdots & O_{N\times 3} & D_{1,r,2}\\ \vdots & \vdots & \ddots & \vdots & \vdots \\ O_{N\times 3} & O_{N\times 3} & \cdots & D_{1,t,M} & D_{1,r,M} \end{array}\right]$$

with

(64) $$B_{1,m}=-2\mathrm{diag}\left(R_{r,1}^{o},R_{r,2}^{o},\ldots ,R_{r,N}^{o}\right)$$

(65) $$D_{1,t,m}=2\left[\begin{array}{c} {\left(u^{o}-{\hat{s}}_{t,m}-R_{r,1}^{o}\rho_{t,m,r,1}\right)}^{T}\\ {\left(u^{o}-{\hat{s}}_{t,m}-R_{r,2}^{o}\rho_{t,m,r,2}\right)}^{T}\\ \vdots \\ {\left(u^{o}-{\hat{s}}_{t,m}-R_{r,N}^{o}\rho_{t,m,r,N}\right)}^{T} \end{array}\right]$$

(66) $$D_{1,r,m}=-2\left[\begin{array}{cccc} {\left(u^{o}-{\hat{s}}_{r,1}-R_{r,1}^{o}\rho_{t,m,r,1}\right)}^{T} & 0_{3\times 1}^{T} & \cdots & 0_{3\times 1}^{T}\\ 0_{3\times 1}^{T} & {\left(u^{o}-{\hat{s}}_{r,2}-R_{r,2}^{o}\rho_{t,m,r,2}\right)}^{T} & \cdots & 0_{3\times 1}^{T}\\ \vdots & \vdots & \ddots & \vdots \\ 0_{3\times 1}^{T} & 0_{3\times 1}^{T} & \cdots & {\left(u^{o}-{\hat{s}}_{r,N}-R_{r,N}^{o}\rho_{t,m,r,N}\right)}^{T} \end{array}\right]$$

From the set of linear equations in (56), the WLS estimate of $\theta^{o}$, denoted by θ, which minimizes $\Delta h_{1}^{T}W_{1}\Delta h_{1}$ can be produced as

(67) $$\theta ={\left(G_{1}^{T}W_{1}G_{1}\right)}^{-1}G_{1}^{T}W_{1}h_{1}$$

where $W_{1}$ represents the weighting matrix and it can be computed by

(68) $$\begin{array}{l} W_{1}={\left[E(\Delta h_{1}\Delta h_{1}^{T})\right]}^{-1}\\ \;={\left[B_{1}Q_{\alpha }B_{1}^{T}+D_{1}\mathrm{cov}(\Delta s-\Delta \hat{s})D_{1}^{T}\right]}^{-1} \end{array}$$

However, to compute $W_{1}$, the unknown target position has to be acquired in advance. To resolve this contradiction, we preliminarily let $W_{1}=I_{MN\times MN}$ and use (67) to compute a least squares estimate of $\theta^{o}$, and then use the estimated $\theta^{o}$ to update $W_{1}$ for another repetition. 

Based on the WLS theorem, it can be deduced that the estimate θ is approximately unbiased and the corresponding covariance matrix can be obtained, given sufficiently small BR measurement noise and transmitter/receiver position error, as

(69) $$\mathrm{cov}(\theta )={\left(G_{1}^{T}W_{1}G_{1}\right)}^{-1}$$

Next, the functional relation between the target position $u^{o}$ and the introduced nuisance parameters $R_{t,1}^{o},R_{t,2}^{o},\ldots ,R_{t,M}^{o}$, is explored to compute the final estimate of target position. To this end, reorganize the functional relation in (4) as

(70) $$2{\left(s_{t,m}^{o}\right)}^{T}u^{o}={\left(u^{o}\right)}^{T}u^{o}+{\left(s_{t,m}^{o}\right)}^{T}s_{t,m}^{o}-{\left(R_{t,m}^{o}\right)}^{2}$$

Denoting the estimation error of θ by Δθ, mathematically we arrive at

(71) $$u^{o}=\theta (1:3)-\Delta \theta (1:3)$$

(72) $$R_{t,m}^{o}=\theta (3+m)-\Delta \theta (3+m)$$

Putting (71), (72) into the right side of (70) and $s_{t,m}^{o}={\hat{s}}_{t,m}-(\Delta s_{t,m}-\Delta {\hat{s}}_{t,m})$ into the both sides, we have after ignoring second-order error terms,

(73) $$\begin{array}{l} 2{\hat{s}}_{t,m}^{T}u^{o}=\theta {\left(1:3\right)}^{T}\theta (1:3)-\theta {\left(m+3\right)}^{2}+{\hat{s}}_{t,m}^{T}{\hat{s}}_{t,m}\\ \;-2\theta (1:3)\Delta \theta (1:3)+2\theta (m+3)\Delta \theta (m+3)+2{\left(u^{o}-{\hat{s}}_{t,m}\right)}^{T}(\Delta s_{t,m}-\Delta {\hat{s}}_{t,m}) \end{array}$$

The final estimate of target position should satisfy (73) and meanwhile retain as close as possible to the estimated values of target position in θ. In line with this principle, one has the following set of equations

(74) $$G_{2}u^{o}=h_{2}+\Delta h_{2}$$

where

(75) $$G_{2}=\left[\begin{array}{c} I_{3\times 3}\\ 2s_{t,1}^{T}\\ 2s_{t,2}^{T}\\ \vdots \\ 2s_{t,M}^{T} \end{array}\right]$$

(76) $$h_{2}=\left[\begin{array}{c} \theta (1:3)\\ \theta {\left(1:3\right)}^{T}\theta (1:3)-\theta {\left(1+3\right)}^{2}+{\hat{s}}_{t,1}^{T}{\hat{s}}_{t,1}\\ \theta {\left(1:3\right)}^{T}\theta (1:3)-\theta {\left(2+3\right)}^{2}+{\hat{s}}_{t,2}^{T}{\hat{s}}_{t,2}\\ \vdots \\ \theta {\left(1:3\right)}^{T}\theta (1:3)-\theta {\left(M+3\right)}^{2}+{\hat{s}}_{t,M}^{T}{\hat{s}}_{t,M} \end{array}\right]$$

(77) $$\Delta h_{2}=B_{2}\Delta \theta +D_{2}(\Delta s-\Delta \hat{s})$$

(78) $$B_{2}=\left[\begin{array}{ccccc} -I_{3\times 3} & 0_{3\times 1} & 0_{3\times 1} & \cdots & 0_{3\times 1}\\ -2\theta (1:3) & 2\theta (1+3) & 0 & \cdots & 0\\ -2\theta (1:3) & 0 & 2\theta (2+3) & \cdots & 0\\ \vdots & \vdots & \vdots & \ddots & \vdots \\ -2\theta (1:3) & 0 & 0 & \cdots & 2\theta (M+3) \end{array}\right]$$

(79) $$D_{2}=\left[\begin{array}{cc} O_{3\times 3M} & O_{3\times 3N}\\ 2\mathrm{diag}\left\{{\left(u^{o}-{\hat{s}}_{t,1}\right)}^{T},{\left(u^{o}-{\hat{s}}_{t,2}\right)}^{T},\ldots ,{\left(u^{o}-{\hat{s}}_{t,M}\right)}^{T}\right\} & O_{3M\times 3N} \end{array}\right]$$

Invoking the WLS theorem again, one has the solution of target position, denoted by **u**, from (74) as

(80) $$u={\left(G_{2}^{T}W_{2}G_{2}\right)}^{-1}G_{2}^{T}W_{2}h_{2}$$

where $W_{2}$ is the weighting matrix and it is determined by

(81) $$\begin{array}{l} W_{2}={\left[E(\Delta h_{2}\Delta h_{2}^{T})\right]}^{-1}\\ \;=[B_{2}\mathrm{cov}(\theta )B_{2}^{T}+D_{2}\mathrm{cov}(\Delta s-\Delta \hat{s})D_{2}^{T}+B_{2}{\left(G_{1}^{T}W_{1}G_{1}\right)}^{-1}G_{1}^{T}W_{1}D_{1}\mathrm{cov}(\Delta s-\Delta \hat{s})D_{2}^{T}\\ \;{+D_{2}\mathrm{cov}(\Delta s-\Delta \hat{s})D_{1}^{T}W_{1}G_{1}{\left(G_{1}^{T}W_{1}G_{1}\right)}^{-1}B_{2}^{T}]}^{-1} \end{array}$$

But as presented in (81), the unknown target position is required in the computation of $W_{2}$. Herein, to circumvent this dilemma, we preliminarily exploit the target position estimate contained in θ to form $W_{2}$ and use (80) to estimate target position. After that we can utilize the estimated target position to update $W_{2}$ for another repetition. 

From the WLS theorem, the covariance matrix of **u** can be approximated, given sufficiently small BR measurement noise and transmitter/receiver position error, as

(82) $$\mathrm{cov}(u)={\left(G_{2}^{T}W_{2}G_{2}\right)}^{-1}$$

### 4.2. Performance Analysis

As mentioned above, the CRLB traces out a lower bound for minimum possible variance that an unbiased estimator can achieve. Next, we will analyze the efficiency of the proposed solution by comparing its covariance matrix with the benchmark, i.e., CRLB. For derivation simplicity, we would compare their inverse, rather than directly compare the two separately. The CRLB has been presented in (33). By invoking the matrix inversion lemma [32] to (33) and using the definitions of **X** and **Y**, we have after mathematical simplifications,

(83) $$CRLB_{c}{\left(u^{o}\right)}^{-1}={\left(\frac{\partial r^{o}}{\partial u^{o}}\right)}^{T}Q_{r}^{-1}\left(\frac{\partial r^{o}}{\partial u^{o}}\right)-{\left(\frac{\partial r^{o}}{\partial u^{o}}\right)}^{T}Q_{r}^{-1}\left(\frac{\partial r^{o}}{\partial s^{o}}\right){\overset{⌣}{Z}}^{-1}{\left(\frac{\partial r^{o}}{\partial s^{o}}\right)}^{T}Q_{r}^{-1}\left(\frac{\partial r^{o}}{\partial u^{o}}\right)$$

where the expression of $\overset{⌣}{Z}$ has been given in (32).

On the other hand, using (82), (81), (69), (68) and (52) successively, we can reformulate the inverse of cov(u) as

(84) $$\mathrm{cov}{\left(u\right)}^{-1}=G_{3}^{T}Q_{r}^{-1}G_{3}-G_{3}^{T}Q_{r}^{-1}G_{4}{\bar{Z}}^{-1}G_{4}^{T}Q_{r}^{-1}G_{3}$$

where $G_{3}=B_{1}^{-1}G_{1}B_{2}^{-1}G_{2}$, $G_{4}=B_{1}^{-1}D_{1}$, and $\bar{Z}=Q_{s}^{-1}+G_{4}^{T}Q_{r}^{-1}G_{4}+G_{0}^{T}{\left(G_{c}Q_{c}G_{c}^{T}+Q_{rc}\right)}^{-1}G_{0}$.

Comparing (83) with (84), we observe that $CRLB_{c}{\left(u^{o}\right)}^{-1}$ and $\mathrm{cov}{\left(u\right)}^{-1}$ are identical in structure. Next, we proceed to prove their equivalency under the following conditions: 

(C1) $\left\|\Delta s_{t,m}\|\ll \|c_{k}^{o}-s_{t,m}^{o}\right\|$, $\left\|\Delta s_{t,m}\|\ll \|s_{t,m}^{o}-s_{r,n}^{o}\right\|$, $\left\|\Delta s_{r,n}\|\ll \|c_{k}^{o}-s_{r,n}^{o}\right\|$, $\left\|\Delta s_{r,n}\|\ll \|s_{t,m}^{o}-s_{r,n}^{o}\right\|$, and $\left\|\Delta c_{k}\|\ll \|c_{k}^{o}-s_{t,m}^{o}\right\|$, $\left\|\Delta c_{k}\|\ll \|c_{k}^{o}-s_{r,n}^{o}\right\|$, for k=1,2,…,K, m=1,2,…,M and n=1,2,…,N;

(C2) $\left\|\Delta r_{m,n}\|\ll \|u^{o}-s_{t,m}^{o}\right\|$, $\left\|\Delta r_{m,n}\|\ll \|u^{o}-s_{r,n}^{o}\right\|$, $\left\|\Delta r_{m,n}\|\ll \|s_{t,m}^{o}-s_{r,n}^{o}\right\|$, and $\|\Delta s_{t,m}-\Delta {\hat{s}}_{t,m}\|\ll$ $\left\|u^{o}-s_{t,m}^{o}\right\|$, $\left\|\Delta s_{r,n}-\Delta {\hat{s}}_{r,n}\|\ll \|u^{o}-s_{r,n}^{o}\right\|$ for m=1,2,…,M and n=1,2,…,N;

The condition C1 implies the transmitter/receiver position error and the calibration target position error are negligibly small compared with the range between the calibration target and the transmitter/receiver. The condition C2 implies the BR measurement noise and the error in the refined transmitter/receiver position are negligibly small compared to the range between the calibration target and the transmitter/receiver. Using the conditions C1 and C2, we obtain, after some involved algebraic manipulations, that

(85) $$G_{3}=\frac{\partial r^{o}}{\partial u^{o}},G_{4}=-\frac{\partial r^{o}}{\partial s^{o}},G_{0}=-\left(\frac{\partial r_{c}^{o}}{\partial s^{o}}\right),G_{c}=-\left(\frac{\partial r_{c}^{o}}{\partial c^{o}}\right)$$

By this point, we can draw the conclusion that

(86) $$\mathrm{cov}{\left(u\right)}^{-1}=CRLB_{c}{\left(u^{o}\right)}^{-1}$$

That is, the proposed solution accomplishes the CRLB accuracy if the two conditions C1 and C2 are satisfied. In reality, localization scenarios, which satisfy the conditions C1 and C2, are not rare. These two conditions can be satisfied if the unknown target and the calibration targets are far from the transmitters and receivers, if not these conditions can still be satisfied if the BR measurement noise and the transmitter/receiver/calibration target position errors are sufficiently small. 

## 5. Simulation Results

In this section, the efficiency and superiority of the proposed solution will be corroborated through Monte Carlo simulations. Amiri’s method presented in [14], which does not consider the transmitter and receiver position error and Zhao’s method proposed in [22], which considers the statistical distributions of transmitter/receiver position error but does not use any calibration targets, are chosen as references for comparison. The exact positions of transmitters/receivers/calibration targets are the same as those in Table 1. Localization accuracy is quantitatively evaluated using root mean squares error (RMSE), which comes from 1000 independent Monte Carlo runs. In each run, the zero-mean Gaussian random errors with covariance matrices $Q_{r}=\sigma_{r}^{2}V_{r}$, $Q_{rc}=\sigma_{\mathrm{rc}}^{2}V_{rc}$, $Q_{s}=\sigma_{s}^{2}V_{s}$ and $Q_{c}=\sigma_{c}^{2}V_{c}$ are added to the BRs from unknown target, BRs from calibration targets, actual transmitter and receiver positions, and actual calibration target positions, respectively, in order to simulate a real localization scenario. The setting of $V_{r}$, $V_{rc}$, $V_{s}$ and $V_{c}$ are also the same as that in Example 1. 

**Figure 4:**
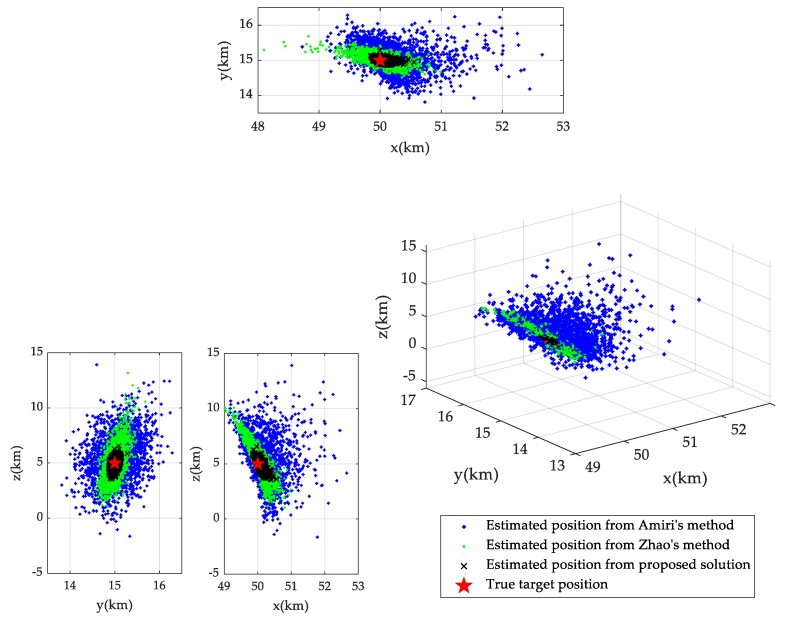
Scatter plots of estimated positions from different methods.

First of all, in order to intuitively show the difference between target localization with and without the use of calibration targets, we plot in Figure 4 the estimated target positions from each Monte Carlo run, which forms a scatter plot for target position estimation. For comparison, the scatterplots of Amiri’s method and Zhao’s method are also plotted. The transmitter/receiver position error level is set to $\sigma_{s}=20\;m$, the noise level of BR measurements from unknown target and calibration targets is set as $\sigma_{r}=\sigma_{\mathrm{rc}}=10\;m$, and the calibration target position error level is set to be $\sigma_{c}=10\;m$. The true position of the unknown target is $u^{o}={\left[50000,15000,5000\right]}^{T}m$, which is marked with red pentagram in Figure 4 for comparison. By comparing the scatterplots of the methods, we find that with the use of calibration targets, the scattered dots of target position estimation are more closely around the target’s true position, which intuitively illustrates the performance gain from the use of calibration targets. Without the use of calibration targets, considering the statistical distributions of transmitter/receiver position error can also reduce dispersion of estimated target position dots to some extent, but compared to using calibration targets, this degree of reduction in dispersion is not sufficiently impressive.

**Figure 5:**
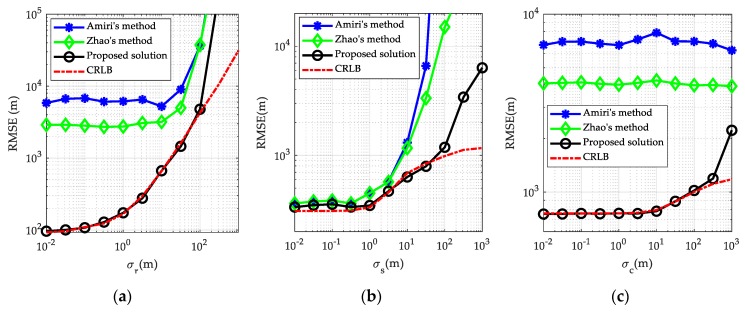
Comparison of the RMSEs among different localization methods in the far-field case: (**a**) with different BR measurement noise level $\sigma_{r}$ and $\sigma_{s}=20\;m$, $\sigma_{c}=10\;m$; (**b**) with different transmitter/receiver position error level $\sigma_{s}$ and $\sigma_{r}=10\;m$, $\sigma_{c}=10\;m$; (**c**) with different calibration target position error level $\sigma_{c}$ and $\sigma_{r}=10\;m$, $\sigma_{s}=20\;m$.

Now, in order to quantitatively evaluate the localization accuracy of the methods, we calculate the RMSE of the proposed solution under different error or noise conditions, and compare it with Amiri’s method, Zhao’s method, as well as the CRLB. As mentioned in Section 4.2, the localization accuracy of the proposed solution is related to the distance between the target and MPR system. Hence, in order to achieve a more comprehensive insight on the performance of the proposed solution, we consider two cases, i.e., the near-field case where the target is close to the MPR system, and the far-field case where the target is far away from the MPR system. The exact positions of transmitters/receivers/calibration targets remain the same as before. We first address the far-field target, whose position is set to $u^{o}={\left[120000,120000,12000\right]}^{T}m$. The results are presented in Figure 5.

Figure 5a plots the RMSE curves of the methods versus the BR measurement noise level. It shows that the localization RMSE of the proposed solution matches the CRLB very well and is about an order of magnitude lower than that of Amiri’s method and Zhao’s method at a low-to-moderate BR measurement noise level. Although it deviates from the CRLB when the BR measurement noise level is large, it is still much smaller than that of other two methods. The deviation from the CRLB, known as the thresholding phenomenon, is due to the ignored second order error terms in the design of the solution, which is invalid for large error levels. Owing to considering the statistical distributions of the transmitter/receiver position error, the RMSEs produced by Zhao’s method is generally lower than that by Amiri’s method. But compared with the use of the calibration targets in the proposed solution, the localization accuracy improvement brought by the consideration of transmitter/receiver position error in Zhao’s method is not so significant. Figure 5b gives the RMSE curves of the methods versus the transmitter/receiver position error level. It can be seen that, the superiority of the proposed solution in localization accuracy is mainly reflected at moderate to high transmitter/receiver position error level. When the transmitter/receiver position error is small, the localization accuracy of the proposed solution and the other two methods is comparable. This again agrees very well with the theoretical performance in Section 3. Figure 5c compares the RMSEs from the methods with respect to different calibration target position error levels. As is illustrated in Figure 5c, the proposed solution always offers a remarkable advantage over the other two methods at different calibration target position error level, even when the calibration target position error is extremely large. This is in agreement with the previous simulation results for the CRLB in Section 3.

**Figure 6:**
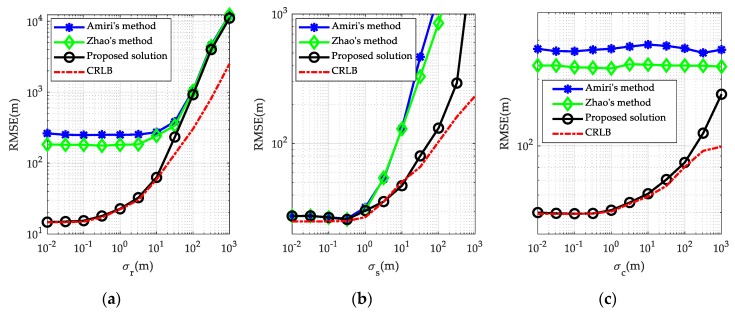
Comparison of the RMSEs among different localization methods in the near-field case: (**a**) with different BR measurement noise level $\sigma_{r}$ and $\sigma_{s}=20\;m$, $\sigma_{c}=10\;m$; (**b**) with different transmitter/receiver position error level $\sigma_{s}$ and $\sigma_{r}=10\;m$, $\sigma_{c}=10\;m$; (**c**) with different calibration target position error level $\sigma_{c}$ and $\sigma_{r}=10\;m$, $\sigma_{s}=20\;m$.

Next, the same set of simulations was repeated for a near-field target, whose position is set to be $u^{o}={\left[12000,1200,1200\right]}^{T}m$. The results are provided in Figure 6, from which we observe that the proposed solution still performs much better than the other methods. However, comparing with the corresponding results in Figure 5, we find the localization accuracy for near-field target is generally better than a far-field target, given the same noise and error levels. One reason may be that, when the target is close to the MPR system, the transmitters/receivers are far apart relative to the distance between the target and the MPR system. Thus, the localization geometry would become more regular and the corresponding geometric dilution of precision (GDOP) value would be smaller compared to the far-field case. However, on the other hand, comparing the thresholding values in Figure 5 and Figure 6 indicates that the RMSE curves for the near-field target deviate from the CRLB at smaller values than those for the far-field target. This phenomenon is consistent with the analysis under (83) that the equivalency between the estimate variance and the CRLB is more affected by the BR measurement noises when the target is close to the MPR system.

**Figure 7:**
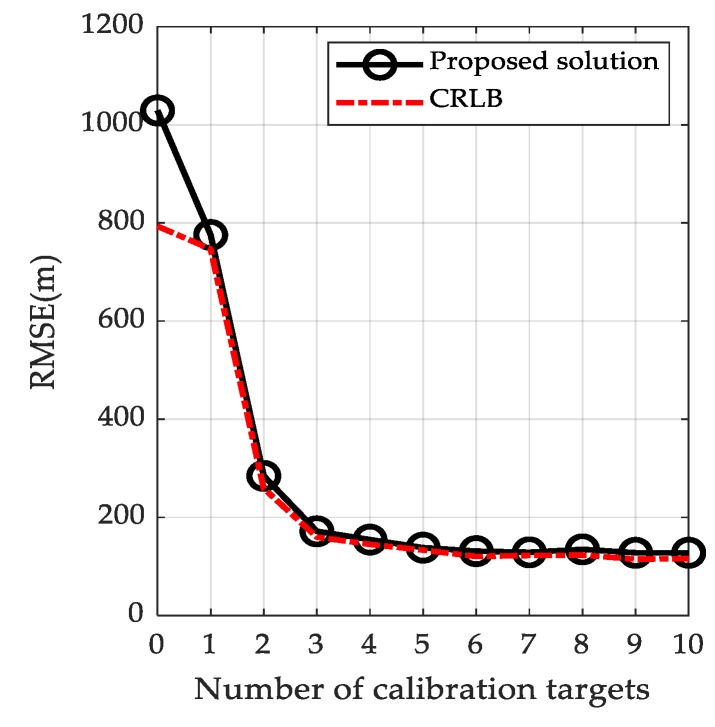
Localization accuracy versus the number of calibration targets.

At an intuitive level, the more calibration targets are used, the better the localization accuracy is. In what follows, we will quantitatively analyze the effect of number of calibration targets on the localization accuracy by varying the number of calibration targets from 1 to 10. The positions of the transmitters and receivers remain the same as before. The positions of calibration targets and unknown target are chosen randomly from the 50 km × 40 km × 5 km volume as presented in Figure 2. The simulation results are depicted in Figure 7.

Figure 7 shows the RMSE, as well as the CRLB, versus the number of calibration targets. As expected, when the number of calibration targets is small, the localization accuracy improves significantly as the number of calibration targets increases. However, it is seen that there is no obvious dependence on the number of calibration targets as soon as the number of calibration targets is larger than 3. This indicates that when the number of calibration targets reaches 3, the use of more calibration targets would only increase the computational expense and not remarkably enhance the localization accuracy. Therefore, in the absence of any other consideration, it is reasonable to set the number of calibration targets as 3.

## 6. Conclusions

This paper explores the use of calibration targets with known positions to refine the inaccurate transmitter/receiver positions and thus enhance target localization accuracy in MPR systems. We start our research by evaluating target localization CRLB in the presence of calibration targets, which justifies the potential of calibration targets in enhancing localization accuracy. Then, in order to fulfill this potential, a novel closed-form solution was designed for target localization using BR measurements from the unknown target as well as the calibration targets. The proposed solution was shown both analytically and numerically to attain the CRLB under some mild conditions, and verified to outperform existing methods in terms of localization accuracy. Furthermore, from the view of engineering practice, if the employed calibration targets are off-the-shelf, such as the commercial aircrafts broadcasting an ADS-B signal, the use of calibration targets would bring little added cost or complexity to the MPR system, but could bring a significant enhancement to target localization accuracy. 

## Figures and Tables

**Table 1:** Positions (in meters) of the transmitters, receivers and calibration targets.

TX	$x_{t,m}^{o}$	$y_{t,m}^{o}$	$z_{t,m}^{o}$	RX	$x_{r,n}^{o}$	$y_{r,n}^{o}$	$z_{r,n}^{o}$	Calibration Targets	$x_{c,k}^{o}$	$y_{c,k}^{o}$	$z_{c,k}^{o}$
1	20,000	0	100	1	2000	2000	0	1	10,000	10,000	2500
2	15,000	5000	1000	2	2000	−2000	500	2	15,000	30,000	3000
3	15,000	−5000	2000	3	5000	5000	1000	3	20,000	−10,000	3500
--	--	--	--	4	5000	−5000	1500	--	--	--	--
